# Transcriptional Profiling of Type II Toxin–Antitoxin Genes of *Helicobacter pylori* under Different Environmental Conditions: Identification of HP0967–HP0968 System

**DOI:** 10.3389/fmicb.2016.01872

**Published:** 2016-11-22

**Authors:** María G. Cárdenas-Mondragón, Miguel A. Ares, Leonardo G. Panunzi, Sabino Pacheco, Margarita Camorlinga-Ponce, Jorge A. Girón, Javier Torres, Miguel A. De la Cruz

**Affiliations:** ^1^Unidad de Investigación Médica en Enfermedades Infecciosas y Parasitarias, Hospital de Pediatria, Centro Médico Nacional Siglo XXI, IMSSMexico City, Mexico; ^2^Centre d’Immunologie de Marseille-Luminy, Aix Marseille Université UM2, Inserm, U1104, CNRS UMR7280Marseille, France; ^3^Departamento de Microbiología Molecular, Instituto de Biotecnología UNAMCuernavaca, Mexico; ^4^Centro de Detección Biomolecular, Benemérita Universidad Autónoma de PueblaPuebla, Mexico

**Keywords:** *H. pylori*, HP0967, HP0968, toxin–antitoxin system, environmental cues

## Abstract

*Helicobacter pylori* is a Gram-negative bacterium that colonizes the human gastric mucosa and is responsible for causing peptic ulcers and gastric carcinoma. The expression of virulence factors allows the persistence of *H. pylori* in the stomach, which results in a chronic, sometimes uncontrolled inflammatory response. Type II toxin–antitoxin (TA) systems have emerged as important virulence factors in many pathogenic bacteria. Three type II TA systems have previously been identified in the genome of *H. pylori* 26695: HP0315–HP0316, HP0892–HP0893, and HP0894–HP0895. Here we characterized a heretofore undescribed type II TA system in *H. pylori*, HP0967–HP0968, which is encoded by the bicistronic operon *hp0968–hp0967* and belongs to the Vap family. The predicted HP0967 protein is a toxin with ribonuclease activity whereas HP0968 is an antitoxin that binds to its own regulatory region. We found that all type II TA systems were expressed in *H. pylori* during early stationary growth phase, and differentially expressed in the presence of urea, nickel, and iron, although, the *hp0968–hp0967* pair was the most affected under these environmental conditions. Transcription of *hp0968–hp0967* was strongly induced in a mature *H. pylori* biofilm and when the bacteria interacted with AGS epithelial cells. Kanamycin and chloramphenicol considerably boosted transcription levels of all the four type II TA systems. The *hp0968–hp0967* TA system was the most frequent among 317 *H. pylori* strains isolated from all over the world. This study is the first report on the transcription of type II TA genes in *H. pylori* under different environmental conditions. Our data show that the HP0967 and HP0968 proteins constitute a *bona fide* type II TA system in *H. pylori*, whose expression is regulated by environmental cues, which are relevant in the context of infection of the human gastric mucosa.

## Introduction

*Helicobacter pylori* is a Gram-negative bacterium, member of the Epsilon proteobacteria that colonizes the human gastric mucosa (Marshall and Warren, 1984). A hallmark of *H. pylori* is its ability to survive in the hostile environment found in the stomach, which is attributed to the expression of various virulence factors, such as secretion systems, cytotoxins, flagella, and adhesins. In this regard, the *H. pylori* genome encodes a type IV secretion system, which is responsible for the translocation of the CagA effector protein into host epithelial gastric cells (Amieva and El-Omar, 2008; Tegtmeyer et al., 2011). In addition to CagA, *H. pylori* produces the vacuolating cytotoxin VacA, which is present in all *H. pylori* strains (Palframan et al., 2012). The toxin–antitoxin (TA) systems have emerged as important virulence factors in many pathogenic bacteria (Kedzierska and Hayes, 2016). At present, six types of TA systems have been described (Page and Peti, 2016) among which, the type II is highly prevalent in prokaryote genomes (Makarova et al., 2009). The functionality of these systems has been described to be beneficial in bacterial fitness, persistence, and virulence (Pandey and Gerdes, 2005; Lewis, 2007; Leplae et al., 2011; Kedzierska and Hayes, 2016). These systems are formed by a couple of TA proteins, where the toxin exerts a bacteriostatic effect on bacterial growth and the antitoxin neutralizes the toxin through a protein-protein interaction. A remarkable characteristic of the type II TA system is that the antitoxin acts as a repressor of its own transcription (Yamaguchi and Inouye, 2011; Yamaguchi et al., 2011). In terms of virulence, type II TA systems have been described to be important in the host–pathogen interactions, particularly in Gram-negative bacteria. In uropathogenic *Escherichia coli*, a type II TA module promotes the colonization of the mouse bladder or kidneys (Norton and Mulvey, 2012). The SehAB system was shown to be important for the survival of *Salmonella enterica* serotype Typhimurium in the mesenteric lymphoid nodes of mice (De la Cruz et al., 2013). Also, the RelBE TA system of *Vibrio cholerae* affects biofilm formation and intestinal colonization (Wang et al., 2015). Moreover, in both *Leptospira interrogans* and *Rickettsia* spp., type II TA mutants were affected in their induction of apoptosis in eukaryotic cells (Audoly et al., 2011; Komi et al., 2015). Thus, a growing body of evidence suggests that these emerging virulence factors display functional and regulatory roles in the expression of multiple virulence traits.

Three type II TA systems have been structurally and functionally characterized in *H. pylori* 26695: HP0315–HP0316, HP0892–HP0893, and HP0894–HP0895 (Han et al., 2011, 2013; Kwon et al., 2012; Pathak et al., 2013; Im et al., 2014). These TA systems belong to the Vap (virulence-associated protein) family, found in Bacteria and Archaea (Makarova et al., 2006). However, no transcriptional analyses have been reported for these TA systems in *H. pylori* under environmental conditions such as acidic pH or in the presence of urea, nickel, and iron, which affect the expression of virulence factors in this bacterium (Sachs et al., 2003; Haley and Gaddy, 2015; Keilberg and Ottemann, 2016).

In this work we identified and characterized a new type II TA system (HP0967–HP0968) in the genome of *H. pylori* strain 26695. Whilst HP0967 turned out to be a toxin with ribonuclease activity, HP0968 was found to work as an antitoxin that bound to its own regulatory region. Compared to the other three known type II TA systems, the *hp0968–hp0967* system was highly expressed when *H. pylori* 26695 was grown in *Brucella* broth, particularly during the early stationary phase. Interestingly, metals such as nickel and iron repressed the expression of *hp0968–hp0967*, while urea had a moderate positive effect. Transcription of *hp0968–hp0967* was highly induced in a mature biofilm and when *H. pylori* was interacting with AGS epithelial cells, whereas the other type II TA systems showed a weaker induction, suggesting that particularly this TA system might be activated in the environment of the host gastric mucosa prior to host colonization. The presence of antibiotics such as kanamycin and chloramphenicol dramatically boosted the transcription of *hp0968–hp0967*. The *hp0968–hp0967* genes were present in both clinical isolates and in *H. pylori* strains whose genomes are publicly available. Our data show that HP0967 and HP0968 proteins form a type II TA system whose expression activates known virulence factors of *H. pylori*. This is the first report about regulation of transcription of *H. pylori* type II TA systems under different environmental conditions.

## Materials and Methods

### Bacterial Strains and Culture Conditions

*Helicobacter pylori* 26695 (Tomb et al., 1997) was grown for 2 days on blood agar culture plates containing 10% defibrinated sheep blood at 37°C under microaerophilic conditions, and a bacterial suspension was prepared in *Brucella* broth and adjusted to an optical density of 0.1 at 600 nm. *H. pylori* was grown at 37°C for 24 h (exponential phase) and 48 h (stationary phase) in *Brucella* broth supplemented with 10% fetal bovine serum (FBS; Invitrogen). In addition, *Brucella* broth adjusted to pH 5.5 or containing either urea [5 μM CO(NH_2_)_2_], nickel [250 μM NiCl_2_] or iron [150 μM (NH_4_)_2_Fe(SO_4_)_2_⋅6H_2_O], were tested as previously described (Contreras et al., 2003; Wen et al., 2003; Vannini et al., 2014). In all cases, the bacteria were incubated at 37°C with shaking and samples were collected 48 h post-inoculation for RNA extraction. Fold-change transcription was determined by calculating the relative expression of TA genes under different environmental conditions as compared to bacteria growing in *Brucella* broth.

### Cloning and Purification of HP0967 and HP0968 Proteins

For cloning of *hp0967* and *hp0968* coding regions, specific primers (**Table 1**) containing the *Nco*I (5′)/*Hind*III (3′) restriction sites were used to obtain PCR products, which were digested with *Nco*I and *Hind*III and then ligated into pBAD-*Myc*-HisA previously digested with the same restriction enzymes. HP0967-*Myc*-His_6_ and HP0968-*Myc*-His_6_ proteins were purified with Ni-nitrilotriacetic acid resin. Briefly, *E. coli* BL21(DE3) carrying the pBAD-HP0967-*Myc*-His_6_ and pBAD-HP0968-*Myc*-His_6_ plasmids were grown to mid-logarithmic phase. L(+)-arabinose (Sigma-Aldrich) was added at final concentration of 0.1%, and the bacteria were grown at 6 h at 30°C. The cells were then pelleted by centrifugation, resuspended in PBS buffer and ruptured by sonication. The suspension was centrifuged and the supernatant was filtered through a Ni-nitrilotriacetic acid agarose column (QIAExpress, Qiagen) pre-equilibrated with PBS buffer. After extensive washing with binding buffer containing 50 μM imidazole (100 ml), the bound protein was eluted with 500 μM imidazole. Fractions were analyzed by SDS-PAGE. Protein concentration was determined by the Bradford procedure. Aliquots of the purified protein were stored at -70°C.

**Table 1:** Primers used in this study.

Primer	Sequence (5′–3′)	Target gene	RE
**For qPCR**			
hp0315-5′	CGGAGAACCCTACAATAAAGCC	*hp0315*	
hp0315-3′	TCCCTTGAGTCCATTCAAACCC		
hp0316-5′	CGCGTTAAATGAACTCTTGC	*hp0316*	
hp0316-3′	ATGCCCCAAACAACAATCTC		
hp0892-5′	AATGCTGACGATTGAAACCAG	*hp0892*	
hp0892-3′	TTTAAACCGCCTTTGAGTGG		
hp0893-5′	CCAAACACCACCAACAAAGA	*hp0893*	
hp0893-3′	GCGCTTTAAATTGGAGTGCT		
hp0894-5′	CCGCTAGATCCACAATTTCAA	*hp0894*	
hp0894-3′	TAAGGGGTGTGGGTGGATTA		
hp0895-5′	GGCATGAGATCCCAAACATT	*hp0895*	
hp0895-3′	TCAAACCCATTCAAAAGCAA		
hp0967-5′	CATGGCTTTAAATGGCAACA	*hp0967*	
hp0967-3′	CCGGCATTAAATCGTTGTTT		
hp0968-5′	TAGTCTTTACGCCCGCTCA	*hp0968*	
hp0968-3′	GGATTTCACATGCTCGCTTT		
cagA-5′	AGCAAAAAGCGACCTTGAAA	*cagA*	
cagA-3′	AGCCAATTGCTCCTTTGAGA		
vacA-5′	AAGCACCATTTGCCTTTGAC	*vacA*	
vacA-3′	CGTTCAATTTCAGCGTGCTA		
16S-5′	GTGTGGGAGAGGTAGGTGGA	*16S*	
16S-3′	GTTTAGGGCGTGGACTACCA		
**For gene cloning**			
HP0967-*Nco*I-5′	AGTTA*CCATGG*ATGCTGTAACTTTTGATCTTGAC	*hp0967*	*Nco*I
HP0967-*Hind*III-3′	GGG*AAGCTT*TAAAACAATCTTGATAGCCGGCATTAAATC		*Hind*III
HP0968-*Nco*I-5′	ATTTC*CCATGG*TAGCTTTAGAAATTTATATTG	*hp0968*	*Nco*I
HP0968-*Hind*III-3′	GGG*AAGCTT*CAACCCATGCGTTTGAGAATAAAACAATTTC		*Hind*III
**For EMSA**			
HP0968-5′	GCTTTATCGCTCTTTTTGGGATTGC	*hp0968*	
HP0968-3′	CAATATAAATTTCTAAAGCGAGCATG		


*Italic letters indicate the respective restriction enzyme site in the primer. RE, restriction enzyme for which a site was generated in the primer.*

### Electrophoretic Mobility Shift Assay (EMSA)

Electrophoretic mobility shift assay (EMSA) experiments were performed as previously described (De la Cruz et al., 2007, 2013). Specific primers (**Table 1**) were used to amplify the *hp0968–hp0967* promoter region by PCR. The PCR product (100 ng) was mixed with increasing concentrations of HP0968-*Myc*-His_6_ at room temperature in the presence of the binding buffer 10X (400 μM HEPES, 80 μM MgCl_2_, 500 μM KCl, 10 μM DTT, 0.5% NP40, and 1 μg/ml BSA). The reactions were incubated during 30 min at room temperature and then separated in 6% SDS-PAGE gels in Tris-Borate-EDTA buffer. The DNA bands were visualized with ethidium bromide staining.

### Ribonuclease Activity Assay

HP0967-*Myc*-His_6_ protein (0, 0.6, and 1.2 μM, respectively) was incubated with 1 μg of total *H. pylori* RNA in the presence of 50 μM Tris (pH 8.0) and 10 μM MgCl_2_ in a final reaction volume of 20 μl. When necessary, HP0968-*Myc*-His_6_ protein was added to analyze the antagonic effect of the antitoxin on the toxin activity. The reaction was allowed to proceed for 30 min at 25°C, and the samples were then loaded in a bleach agarose gel 2% as previously described (Aranda et al., 2012). RNA was visualized with ethidium bromide staining.

### RNA Isolation and Quantitative RT-PCR (qRT-PCR)

Total RNA was extracted from the different culture conditions using the hot phenol method (Jahn et al., 2008). Purification of RNA, and qRT-PCR were performed as previously reported (Ares et al., 2016). Briefly, DNA was removed with TURBO DNA-free (Ambion, Inc.) and the quality of RNA was assessed using a NanoDrop (ND-1000; Thermo Scientific) and a bleach agarose gel 2% as previously described (Aranda et al., 2012). cDNA was prepared using 1 μg of RNA, random hexamer primers (0.2 μg/μl), and M-MulV-RT (20 U/μl, reverse transcriptase of Moloney Murine leukemia Virus; Thermo Scientific). Specific primers were designed with the Primer3Plus software^1^ and are listed in **Table 1**. The absence of contaminating DNA was controlled by lack of amplification products after 35 qPCR cycles. Control reactions with no template (water) and minus-Reverse Transcriptase were run with all reactions. 16S rRNA (HPrrnA16S) was used as a reference gene for normalization and the relative gene expression was calculated using the 2^-ΔCt^ method (Livak and Schmittgen, 2001).

### TA Gene Expression during *H. pylori* Infection of AGS Cells

AGS gastric epithelial cells were grown to about 75% confluence in RPMI-1640 medium containing 10% FBS, and washed thrice with PBS before adding fresh RPMI media with 10% FBS. *H. pylori* 26695 was grown in *Brucella* broth for 24 h, suspended in RPMI, and added to the AGS cell culture at a multiplicity of infection (MOI) of 100. Infected cells were incubated at 37°C under microaerophilic conditions for 0 and 6 h, and bacteria were recovered. RNA was extracted from bacteria to determine gene expression. Fold-change in gene transcription was determined by calculating the relative expression of TA genes with respect to bacteria at time 0 of infection.

### Biofilm Formation Assay on Abiotic Surface and TA Gene Expression

Adhesion to abiotic surface (polystyrene) was analyzed using 96-well plates as previously described (Ares et al., 2016). *H. pylori* 26695 was grown for 2 days on blood agar culture plates containing 10% defibrinated sheep blood at 37°C under microaerophilic conditions, and a bacterial suspension was prepared in *Brucella* broth supplemented with 10% decomplemented FBS and adjusted to an optical density of 0.1 at 600 nm. Two-hundred μl of the bacterial suspensions were added per well into 96-well plates in octuplicate and incubated at different times at 37°C under microaerophilic conditions. Unbound bacteria were removed by washing the wells three times with PBS, and bound bacteria were stained with 1% Crystal Violet for 20 min. Wells were thoroughly rinsed thrice with PBS and the dye was solubilized in 100 μl of ethanol 70%. Finally, the amount of extracted Crystal Violet was determined by measuring color absorbance at OD_600_ using a spectrophotometer plate reader (Thermo Scientific).

For determination of TA gene expression, the biofilm assay was performed in 6 well plates (3 ml) and supernatant (planktonic) and adhered (sessile) bacteria were recovered for RNA extraction. Fold-change in gene transcription was determined by calculating the relative expression of TA genes within biofilms (sessile bacteria) as compared to the planktonic bacteria.

### Transcription of TA Genes in the Presence of Antibiotics

*Helicobacter pylori* was grown in *Brucella* broth supplemented with 10% decomplemented FBS at 37°C for 48 h (stationary phase), with gentle shaking under microaerophilic conditions. The antibiotics ampicillin (Ap, 100 μg/mL), kanamycin (Km, 50 μg/mL), chloramphenicol (Cm, 30 μg/mL), or tetracycline (Tc, 10 μg/mL) were added to the liquid culture and incubated for 1 h as previously described (Christensen-Dalsgaard et al., 2010). Antibiotics concentrations were used as reported for *E. coli* and *Salmonella enterica* (Christensen-Dalsgaard et al., 2010; Maisonneuve et al., 2011; Silva-Herzog et al., 2015; Li et al., 2016). Fold-change in gene transcription was determined by calculating the relative expression of TA genes in the presence of each antibiotic as compared to bacteria growing in *Brucella* broth without antibiotics.

### *In silico* Identification of TA Modules

Analyzed sequence data and loci annotations of 260 *H. pylori* genomes were retrieved from the NCBI database^2^ by a series of custom Perl scripts. In addition, the genomes of 57 *Helicobacter* non*-pylori* strains were included in the comparative analysis.

We also selected the sequences from known toxin and antitoxin modules that were classified into super-families as stated previously (Leplae et al., 2011) and compared their homology with previously referred *Helicobacter* genomes. Briefly, each TA protein pair was queried using PSI-BLAST (Altschul et al., 1997) under the following parameters: matrix = BLOSUM62, word size = 3, PSI-BLAST threshold = 0.005, expect threshold = 10, and without filtering low complexity regions. Hits were carefully examined and selected according to their functional annotation.

Furthermore, we employed the Toxin–Antitoxin Data Base (TADB^3^) (Shao et al., 2011) to extend our findings.

### Heatmap Construction

In order to illustrate the presence of every toxin and antitoxin module in all *Helicobacter* genomes, a gene content matrix (“heatmap” function) was built using the R program^4^ v3.2.4. Next, these paired loci were hierarchical clustered (“hclust” function, “ward.D” method) according to their loci-content similarity to build a sidelong dendogram.

## Results

### The *hp0968–hp0967 H. pylori* Genes Code for a New Type II TA System

**FIGURE 1:**
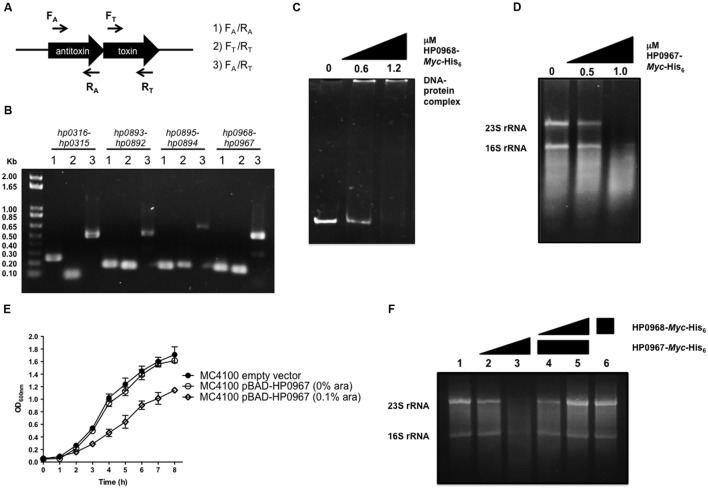
**HP0967–HP0968 is a *bona fide* type II TA system.** **(A)** Schematic representation of the type II TA operons amplified for RT-PCR analysis using specific primers for toxin and antitoxin genes (arrows). **(B)** Qualitative RT-PCR assays carried out with RNA extracted from *Helicobacter pylori* 26695 grown in *Brucella* broth during 48 h. This is a representative result of two independent experiments. **(C)** EMSA exemplifying the binding of HP0968 *Myc*-His_6_ to *hp0968–hp0967* promoter region. DNA-protein complex is indicated in the figure. **(D)** Ribonuclease assay using amounts of HP0967-*Myc*-His_6_ toxin with 1 μg total RNA. 23S rRNA and 16S rRNA are shown in the figure. **(E)** Growth kinetic of wild-type *Escherichia coli* (MC4100) harboring the empty vector pBAD-*Myc*-HisA and the pBAD-HP0967-*Myc*-His_6_ plasmid without and with induction of L-arabinose (ara). Bacterial cultures were grown for 8 h in LB medium at 37°C. **(F)** Ribonuclease inhibition assay using HP0967-*Myc*-His_6_ toxin in presence of HP0968-*Myc*-His_6_ antitoxin with 1 μg of total RNA. Alone RNA (lane 1); RNA with 0.5 (lane 2) and 1.0 μM (lane 3) of HP0967-*Myc*-His_6_ toxin; RNA with 1.0 μM of HP0967-*Myc*-His_6_ toxin and in presence of 0.6 (lane 4) and 1.2 μM (lane 5) of HP0968-*Myc*-His_6_ antitoxin; RNA with 1.2 μM of HP0968-*Myc*-His_6_ antitoxin (lane 6).

Three pairs of type II TA proteins have been described in *H. pylori*: HP0315–HP0316, HP0892–HP0893, and HP0894–HP0895 (Han et al., 2011, 2013; Kwon et al., 2012; Im et al., 2014). Searching for TA-encoding homolog genes of different families, we identified *hp0967*, a gene that codes for a type II toxin belonging to the VapD protein family. Of note, an intergenic region was identified between *hp0967* and *hp0969* genes and was designated herein as antitoxin gene *hp0968*. In contrast, by analyzing the genome sequence of *H. pylori* J99 strain, a homolog of *hp096*8 (*jhp0902*) antitoxin gene was found. The analysis of the *H. pylori* 26695s genome revealed a stop codon in the coding region of *hp0968* gene resulting in a peptide of 19 aminoacids. However, by comparing the nucleotidic sequences of all *H. pylori* strains deposited in the GenBank, no stop codon was found in this gene, suggesting a problem of annotation in the strain 26695. To demonstrate that *hp0968* antitoxin gene is present and transcribed in 26695 strain, we performed RT-PCR experiments using primers that hybridize with TA genes as shown in **Figure 1A**. We found that both *hp0968* and *hp0967* genes were transcriptionally expressed and as expected, they presented a single mRNA similar to other type II TA systems (**Figure 1B**). The analyses of the internal controls for *hp0316–hp0315*, *hp0893–hp0892*, and *hp0895–hp0894* TA genes also showed that they are genetically organized as bicistronic operons (**Figure 1B**).

We also amplified the coding region of both *hp0967* and *hp0968* genes of *H. py*lori 26695 and cloned them in a pBAD-Myc/HisA vector in order to overexpress and purify the proteins. The sequence analysis of the amplified *hp0968* gene revealed that it did not present the stop codon, and that it encodes a functional protein of 93 aminoacids (12.8 kDa). In order to demonstrate the functionality of this antitoxin, EMSA experiments were performed incubating the promoter region of *hp0968–hp0967* with the HP0968-*Myc*-His_6_ protein. The DNA-protein complex was observed at 1.2 μM of HP0968-*Myc*-His_6_ antitoxin (**Figure 1C**). In contrast, the aminoacid sequence of HP0967 presented a PIN domain associated with ribonuclease activity. Total RNA of *H. pylori* 26695 was incubated with 0.5 and 1.0 μM of HP0967-*Myc*-His_6_ and RNA stability was determined. The assay showed that HP0967-*Myc*-His_6_ (1.0 μM) degraded *H. pylori* RNA, suggesting that it is indeed a toxin (**Figure 1D**). In addition, the overexpression of HP0967 toxin affected the growth of *E. coli* (**Figure 1E**). To demonstrate the inhibitory effect of HP0968 antitoxin on HP0967 toxin, we added HP0968-*Myc*-His_6_ protein to the ribonuclease activity assay. Ribonuclease activity of HP0967-*Myc*-His_6_ toxin was inhibited in the presence of HP0968-*Myc*-His_6_ antitoxin (**Figure 1F**). HP0968-*Myc*-His_6_ alone protein did not affect RNA stability. Thus, our data strongly suggests that both HP0967 and HP0968 proteins constitute a new type II TA system of the Vap family in *H. pylori*.

### Environmental Cues Differentially Regulate Expression of *H. pylori* Type II TA Systems

**FIGURE 2:**
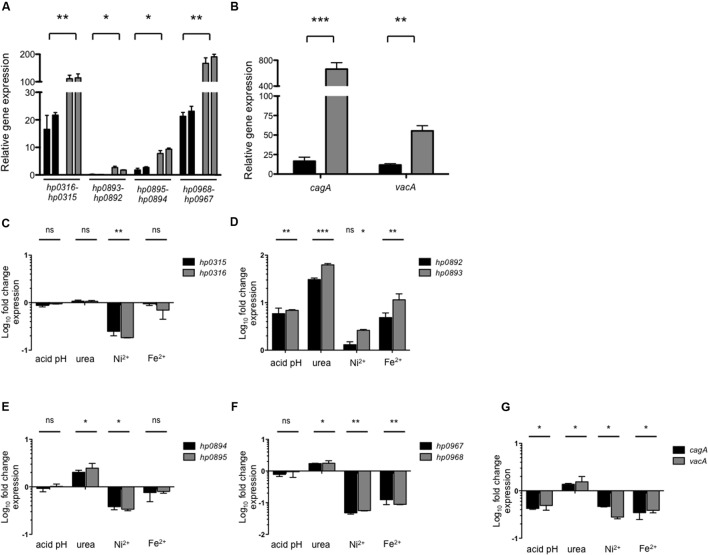
**Environmental cues and expression of type II TA genes.** Expression (qRT-PCR) of the type II TA genes **(A)** and *cagA/vacA* **(B)** of *H. pylori* 26695 in exponential (black bars) and stationary phase (gray bars). Fold-change expression (qRT-PCR) of the type II TA genes **(C–F)** and *cagA/vacA* **(G)** under different environmental conditions with respect to *Brucella* broth at 48 h. Data represent the mean of at least three independent experiments (mean ± SD). ns, not significant; statistically significant ^∗∗∗^*p* < 0.001; ^∗∗^*p* < 0.01; ^∗^*p* < 0.05.

In order to elucidate the environmental cues that may affect expression of type II TA systems we performed qRT-PCR experiments. Relative expression was determined during both exponential (24 h) and stationary phase (48 h). Interestingly, expression of all the type II TA genes was higher during stationary phase than in exponential phase (**Figure 2A**); although *hp0316–hp0315* and *hp0968–hp0967* showed the highest transcriptional levels (**Figure 2A**), and *hp0893–hp0892* and *hp0895–hp0894* were poorly expressed in this growth condition. Similarly, expression of *cagA* and *vacA* was found increased during stationary growth phase (**Figure 2B**), in agreement to previously reported data (Thompson et al., 2003; Boonjakuakul et al., 2005).

Of note, the effect of the environmental cues such as pH, urea, nickel, and iron in transcription of type II TA systems has not been reported for *H. pylori*, although, they are known to affect the expression of other virulence factors (Sachs et al., 2003; Haley and Gaddy, 2015; Keilberg and Ottemann, 2016). We found that during stationary growth phase type II TA genes showed different levels of expression in the presence of the tested cues. While nickel repressed the expression of *hp0316–hp0315* (fivefold) and *hp0895–hp0894* (threefold) (**Figures 2C,E**), all conditions tested triggered expression of *hp0893–hp0892* TA genes (**Figure 2D**). Expression of *hp0968–hp0967* was repressed in the presence of nickel (20-fold) and iron (10-fold), while urea caused a twofold increase in its expression (**Figure 2F**). As reaction controls, the transcription of *cagA* and *vacA* genes was determined under the same growth conditions. We found that transcription of both virulence genes was differentially regulated: urea slightly boosted transcription (1.5-fold), while acidic pH (2.2-fold), nickel (2.7-fold), and iron (2.7-fold) repressed them (**Figure 2G**).

### Biofilm Formation and Contact with Gastric Epithelial Cells Stimulate the Expression of *hp0968–hp0967*

**FIGURE 3:**
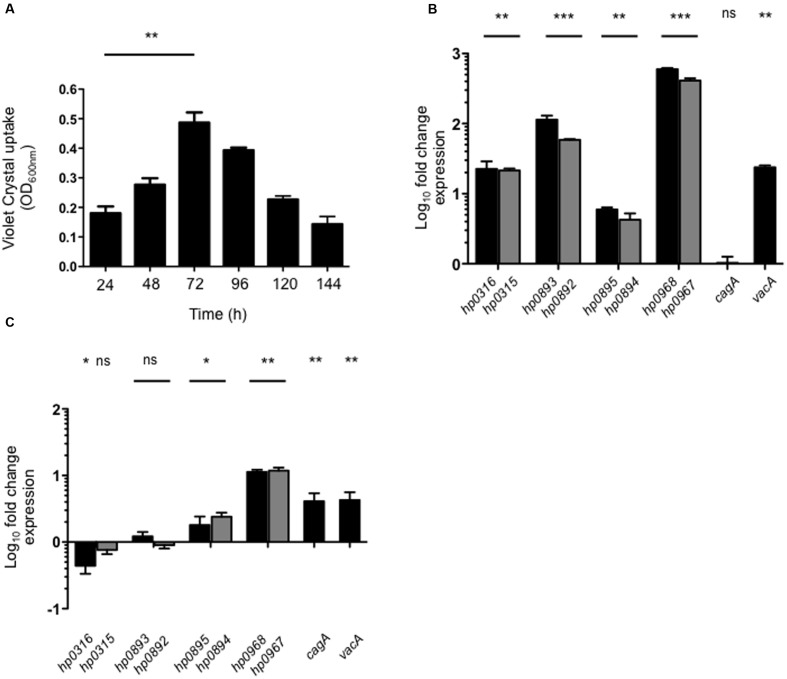
**Expression of type II TA genes in biofilm formation and in AGS cells.** **(A)** Quantification of biofilm formation of *H. pylori* 26695 by measuring Crystal Violet uptake at different times. Fold-change expression (qRT-PCR) of the type II TA genes and *cagA/vacA* in a mature biofilm after 72 h **(B)** and in contact with AGS cells **(C)**. Data represent the mean of at least three independent experiments (mean ± SD). ns, not significant; statistically significant ^∗∗∗^*p* < 0.001; ^∗∗^*p* < 0.01; ^∗^*p* < 0.05.

Type II TA systems are reported to influence biofilm formation (Wang and Wood, 2011; Wen et al., 2014; Kedzierska and Hayes, 2016). We quantitatively analyzed the biofilm formation of *H. pylori* on a polystyrene surface at different time points measuring uptake of Crystal Violet. After 72 h of incubation, a mature biofilm was observed (**Figure 3A**). Thus, in order to evaluate the expression of type II TA genes in a mature biofilm, we determined their expression in both *H. pylori* planktonic and sessile cells at 72 h. Expression of all type II TA genes was found to be induced in biofilms-associated *H. pylori*, as compared to planktonic bacteria (**Figure 3B**). It should be noted that *hp0968–hp0967* showed the highest induction in this environment (500-fold). In a mature biofilm, *vacA* transcription was significantly increased (23-fold), whereas *cagA* expression was not affected (**Figure 3B**). On the other hand, the contact with AGS cells significantly increased transcription of type II TA genes. Of note, *hp0968–hp0967* genes presented the highest levels of expression over the other TA systems, even higher than expression of *cagA* and *vacA* (**Figure 3C**). These data strongly suggest that *hp0968–hp0967* genes might be expressed in the host during stomach colonization.

### The Presence of Antibiotics Affects the Transcription of *H. pylori* Type II TA Genes

**FIGURE 4:**
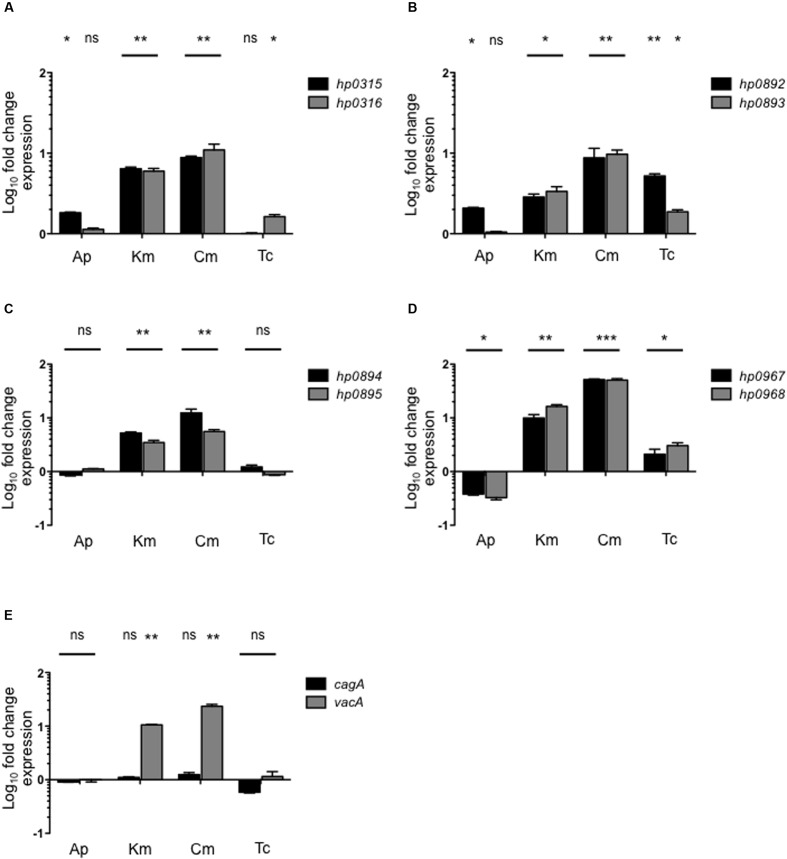
**Effect of antibiotics on type II TA expression.** Fold-change expression (qRT-PCR) of the type II TA genes **(A–D)** and *cagA/vacA* **(E)** in presence of different antibiotics concentrations such as: Ap (Ampicillin, 100 μg/ml), Km (Kanamycin, 30 μg/ml), Cm (Chloramphenicol, 30 μg/ml), and Tc (Tetracycline, 10 μg/ml) by 1 h. Data represent the mean of at least three independent experiments (mean ± SD). ns, not significant; statistically significant ^∗∗∗^*p* < 0.001; ^∗∗^*p* < 0.01; ^∗^*p* < 0.05.

We analyzed the expression of type II TA genes in the presence of antibiotics, and found that kanamycin and chloramphenicol (Km/Cm) increased the expression of *hp0316–hp0315* (6.2/9.9-fold), *hp0893–hp0892* (3.1/9.2-fold), and *hp0895–hp0894* (4.34/9-fold) systems (**Figure 4**). In particular, chloramphenicol, kanamycin, and tetracycline stimulated the expression of *hp0968–hp0967* genes (50-, 12-, and 2.5-fold, respectively) while ampicillin repressed them (2.8-fold) (**Figure 4D**). Interestingly, similar transcriptional profiling was found between the *hp0893–hp0892* and *hp0968–hp0967* genes in presence of chloramphenicol, kanamycin, and tetracycline (**Figures 4B,D**). Concerning the effect of antibiotics on the expression of cytotoxin genes, only *vacA* expression was boosted in the presence of kanamycin (10-fold) and chloramphenicol (23-fold) (**Figure 4E**). These results suggest that type II TA genes may have an important role in the response of *H. pylori* to the presence of antibiotics by *H. pylori*, particularly to chloramphenicol and kanamycin.

### The *hp0968–hp0967* Genes Are Highly Prevalent among *Helicobacter* Strains

**FIGURE 5:**
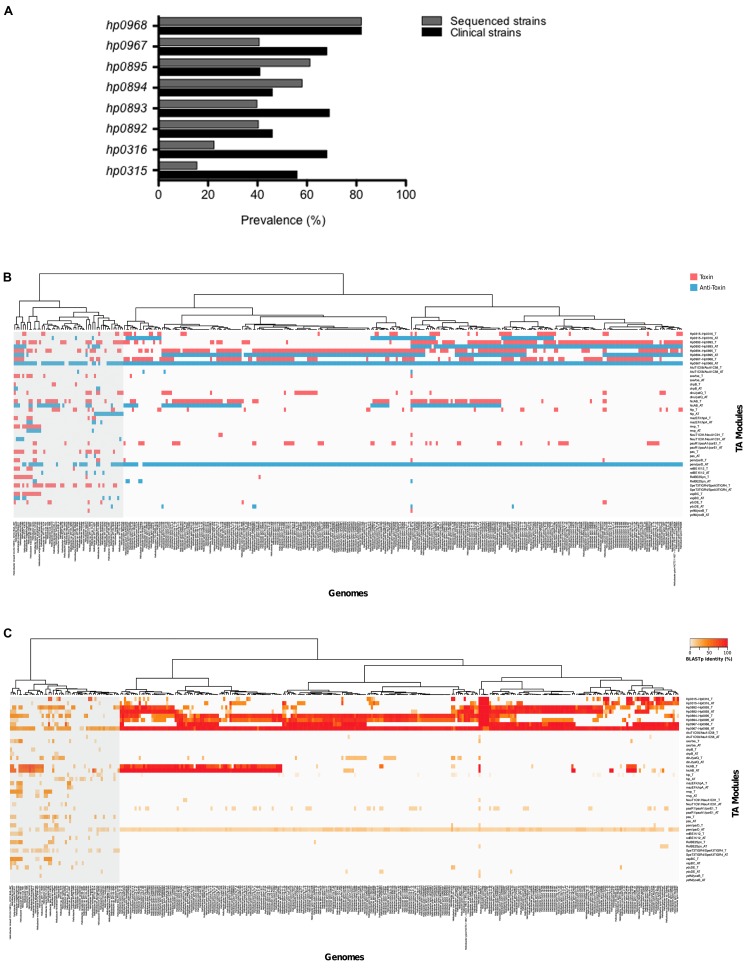
**Prevalence of type II TA genes.** **(A)** The clinical isolates tested (*n* = 114) comprised strains of *H. pylori* with different pathologies. Bars show the prevalence of type II TA genes in clinic (black) and sequenced (gray) strains. The heatmaps and hierarchical clustering of selected genomes for *H. pylori* and *Helicobacter* species showing both the presence **(B)** and identity **(C)** of toxin–antitoxin genes were determined in R (version 3.2.4) using the hclust function with the “ward.D” method. The red and blue colors indicate the presence of the toxin and the anti-toxin gene.

We performed PCR experiments to identify type II TA genes in a collection of 114 clinical *H. pylori* isolates. These bacteria were isolated from patients with diverse pathologies, such as non-atrophic gastritis, premalignant lesions, gastric ulcer, and gastric cancer. Whereas *hp0968–hp0967* was the most prevalent type II TA system in these isolates (in over 70% of all isolates), *hp0895–hp0894* genes were the least prevalent (in less than 50%) (**Figure 5A**). In addition, a Blast search in sequences deposited in GenBank^5^ using sequences of different TA families (included Vap), revealed the presence of type II TA genes in other *H. pylori* strains. TA genes of the Vap family were highly prevalent, presenting high identity and constituting a genetic signature inside *H. pylori* species (**Figure 5B**). In agreement with our findings in the clinical isolates, the *hp0968–hp0967* genes presented the highest prevalence as compared to the other type II TA genes (**Figures 5A–C**). Interestingly, in both groups of *H. pylori* clinical and sequenced strains, the *hp0968* antitoxin gene was more conserved than *hp0967* toxin gene (**Figures 5A–C**).

Since the number of type II TA systems has been associated to bacterial pathogenicity (Georgiades and Raoult, 2011; De la Cruz et al., 2013), we studied the presence of the type II TA systems of different families in other *Helicobacter* species, and found that the presence of type II TA genes varied from one species to another. Species phylogenetically related to *H. pylori* such as *H. acinonychis* (big cats) and *H. cetorum* (marin mammals), contain the four type II TA systems (*hp0316–hp0315*, *hp0893–hp0892*, *hp0895–hp0894*, and *hp0968–hp096*7) and show high identity to the *H. pylori* sequences (**Figures 5B,C**). The presence of TA genes marked a separation of *H. acinonychis* and *H. cetorum* from the rest of *Helicobacter* species and they were grouped together with the *H. pylori* strains.

## Discussion

*Helicobacter pylori* produces a number of virulence-associated proteins such as secretion systems, flagella, adhesins, and cytotoxins that enable successful colonization of its human host. Expression of virulence factors also allows for the survival and persistence of *H. pylori* in the hostile environment found in the stomach (Salama et al., 2013). This persistence is mediated by a myriad of bacterial strategies to evade the immune response, such as detoxifying enzymes for reactive species of oxygen and nitrogen, DNA repair pathways, and natural competence (Wang et al., 2006; Dorer et al., 2011, 2013). In this sense, type II TA systems have emerged as potential virulence factors, not only affecting pathogenicity, but they are also involved in biofilm formation and persistence (Gerdes and Maisonneuve, 2012; Wen et al., 2014; Kedzierska and Hayes, 2016). In this work, we describe the transcriptional profiling of type II TA genes present in the genome of *H. pylori* 26695: *hp0316–hp0315*, *hp0893–hp0892*, *hp0895–hp0894*, and *hp0968–hp0967*, since the *hp0968–hp0967* system had not been identified and it was characterized in this work. These genes are transcriptionally organized as bicistronic operons, which is a hallmark of the type II TA systems. Type II toxins present different biochemical activities such as ribonucleases, kinases, adenylyl- and acetyl-transferases, and act also as inhibitors of protein synthesis and DNA supercoiling (Jiang et al., 2002; Mutschler et al., 2011; Yamaguchi et al., 2011; Harms et al., 2015; Cheverton et al., 2016; Rocker and Meinhart, 2016). In contrast, type II antitoxins are DNA-binding proteins that regulate their own expression and directly interact with their cognate toxins (Yamaguchi and Inouye, 2011; Chan et al., 2016). Despite having more specific functions, antitoxins show more complexity by possessing two functional domains: a DNA-binding domain and a toxin-interaction domain. Our characterization of both *H. pylori* HP0967 and HP0968 proteins revealed that they display ribonuclease and DNA-binding activities, respectively. HP0967 and HP0968 proteins belong to the Vap family, which is the type II TA pair most widespread in Bacteria and Archaea (Pandey and Gerdes, 2005; Sevin and Barloy-Hubler, 2007; Arcus et al., 2011; Jin et al., 2015).

In the stomach, *H. pylori* is exposed to frequent changes in pH, for example to acidic pH when present in the lumen and to neutral pH as it crosses the mucus to interact with epithelial cells. Presumably, these changes alter the expression of genes related to colonization and persistence in its human host. We found that an acidic pH boosted the expression of *hp0893–hp0892*, while no effect was observed for the other type II TA systems, highlighting the importance of this system in the response to the acidic pH, which *H. pylori* frequently finds prior to colonization of the stomach mucosa. Previous reports regarding the effect of acidic pH on the expression of the most thoroughly studied virulence factors *cagA* and *vacA* have been controversial (Karita et al., 1996; Allan et al., 2001; Merrell et al., 2003a; Bury-Mone et al., 2004; Vannini et al., 2014). Our results support the observation that an acidic pH represses both *cagA* and *vacA*, in agreement to the notion that CagA and VacA are produced when *H. pylori* is in close contact to epithelial cells, where the pH is neutral (Salama et al., 2013). Urea, nickel, and iron are components present in the human stomach and it has been suggested that they act as regulatory networks whose purpose is the survival and persistence of *H. pylori* (Haley and Gaddy, 2015). Urea and nickel are interconnected cues because transcription/activity of urease is nickel-dependent (Contreras et al., 2003; Khan et al., 2009). While high concentrations of nickel repress virulence genes (Haley and Gaddy, 2015), the presence of urea is essential for the survival and colonization of *H. pylori* (Nakazawa, 2002; Sachs et al., 2003). We found that urea strongly stimulated the expression of *hp0893–hp0892*, while it had a mild effect in the expression of *hp0895–hp0894* and *hp0968–hp0967*. With the exception of *hp0893–hp0892*, nickel and iron repressed the type II TA systems in *H. pylori*. Our results suggest that the high concentration of urea and low concentrations of both nickel and iron present in the stomach, would induce transcription of type II TA systems in preparation for successful colonization and persistent infection. Our study represents the first report on the transcriptional analysis of type II TA genes in *H. pylori* under several environmental conditions that mimic those prevailing in the gastric mucosa.

Contrasting data have been reported concerning metal regulation of *vacA* and *cagA* (Merrell et al., 2003b; Ernst et al., 2005; Pich et al., 2012; Noto et al., 2013; Vannini et al., 2014). Iron exposure is associated with repression of numerous factors involved in the repertoire of virulence in *H. pylori* (Haley and Gaddy, 2015). The role of nickel on the expression of *vacA* and *cagA* genes has not been previously studied. Transcriptional data showed that both, nickel and iron repressed both *cagA* and *vacA* genes, supporting the notion that the presence of both metals affect the virulence of *H. pylori*.

Biofilm formation is a bacterial strategy to counteract the stress caused by the limitation of nutrients and the effect of antibiotics. It is well-established that type II TA systems are required for the biofilm formation (Wang and Wood, 2011; Wen et al., 2014; Kedzierska and Hayes, 2016). Since environmental and clinical strains of *H. pylori* produce biofilm to persist and colonize both abiotic and biotic surfaces (Cammarota et al., 2012; Percival and Suleman, 2014), we studied the expression of the four type II TA genes in *H. pylori* growing in a mature biofilm. In this condition, the four type II TA systems were induced, supporting the notion that these systems are expressed and may be required for the biofilm formation.

In contact with AGS gastric epithelial cells, transcription of *hp0968–hp0967* was considerably stimulated, presenting values higher than *cagA* and *vacA* cytotoxin genes. These results would support a probable role of *hp0968–hp0967* genes in the context of infection of *H. pylori*.

Kenn Gerdes’s lab has clearly shown that antibiotics such as chloramphenicol, positively affect the expression of TA genes (Christensen et al., 2001; Christensen-Dalsgaard and Gerdes, 2006; Jorgensen et al., 2009; Winther and Gerdes, 2009; Christensen-Dalsgaard et al., 2010). We found that the presence of both chloramphenicol and kanamycin boosted transcription of all type II TA systems in *H. pylori*. In the presence of both kanamycin and chloramphenicol, bacterial translation is inhibited and proteases such as Lon specifically degrades the type II antitoxins, generating an increase in the transcription of TA genes due to the lack of repression of the antitoxins on their own expressions (Christensen et al., 2001; Winther and Gerdes, 2009; Christensen-Dalsgaard et al., 2010). We found that the presence of both kanamycin and chloramphenicol, increased expression of type II TA genes, suggesting a probable role of type II TA systems in the bacterial fitness during biofilm formation and in the presence of antibiotics. In agreement, recent reports show that type II TA modules are necessary for the development of persister cells in the presence of antibiotics (Maisonneuve et al., 2011; Wen et al., 2014; Kedzierska and Hayes, 2016). Current experiments in our group are aimed to further analyze the expression of type II TA systems in persister *H. pylori*.

In most reports, expression of *cagA* and *vacA* has been found to be co-regulated, corroborating the concept that both cytotoxins are required and co-expressed in the context of infection (Yokoyama et al., 2005; Singh et al., 2012; Raghwan and Chowdhury, 2014). However, we found that in a mature biofilm and in the presence of kanamycin or chloramphenicol, transcription of *vacA*, but not *cagA* was significantly increased. In contrast to CagA which is translocated into host cells by the type 4 secretion system, VacA is secreted by a type 5 secretion system (autotransporter) and it can also be located on the bacterial surface (Ilver et al., 2004). Based on the above notions, we suggest that VacA may have a role in bacterium-bacterium interactions, as it occurs during biofilm formation.

In terms of prevalence, *hp0968–hp0967* system was the most prevalent in both clinical and sequenced *H. pylori* strains. Moreover, the *hp0968–hp0967* system was also widely distributed in all *Helicobacter* species. It was recently reported the retention of the antitoxin gene but not the toxin gene in TA modules of *Xanthomonas* genomes (Martins et al., 2016). Both clinical and sequenced *H. pylori* strains revealed the same profile, loss of toxin and retention of antitoxin genes. In *H. pylori* and non-*pylori* genomes deposited in GenBank, *hp0968* antitoxin gene was much more prevalent than *hp0967* toxin. The retention of antitoxin genes in bacterial genomes has been postulated due to the toxin-independent functions of type II antitoxins such as MqsA and DinJ (Wang and Wood, 2011; Hu et al., 2012).

The presence and homology of type II TA genes in *Helicobacter* strains generated two main clusters: *H. pylori* and other *Helicobacter* species, showing that these TA systems represent a DNA signature for *H. pylori.* Moreover, species phylogenetically related to *H. pylori* such as *H. acinonychis* and *H. cetorum* were separated from the rest of *Helicobacter* species and grouped with *H. pylori* strains, supporting the concept that TA genes are part of the prokaryotic mobilome and that they are involved in the bacterial evolution (Koonin and Wolf, 2008). Searching type II TA genes by using Toxin–Antitoxin Data Base (TADB^3^) we found two TA systems for the *H. pylori* strains 26695 and J99 (HP0892–HP0893 and HP0894–HP0895). In the case of other *Helicobacter* species, *H. hepaticus* was the only species showing one TA system (HH0272–HH0273), which clearly indicates the need for an update and construction of a new database that integrates more bacterial genomes.

It is worth noticing that *hp0968–hp0967* genes presented the same transcriptional profile than *cagA* and *vacA* under growth in urea, nickel, iron and in contact with AGS cells. These findings together with the high prevalence of this TA system in *H. pylori* strains, suggest that the *hp0968–hp0967* may represent a very important virulence factor. We cannot discard a probable virulence role for the other type II TA system, since these genes presumably display redundant and specific functions. The generation of null mutants will help to elucidate the role of type II TA genes in different phenotypes such as biofilm formation, persistence and mainly in the virulence of *H. pylori*.

## Author Contributions

Conceived and designed the experiments: MDC. Performed the experiments: MC-M, MA, LP, SP. Analyzed the data: MDC, MC-M, MA, MC-P, JG, JT. Wrote the paper: MDC, JG, JT.

## Conflict of Interest Statement

The authors declare that the research was conducted in the absence of any commercial or financial relationships that could be construed as a potential conflict of interest.

## Footnotes

1 http://www.bioinformatics.nl/cgi-bin/primer3plus/primer3plus.cgi/

2 ftp://ftp.ncbi.nlm.nih.gov/genomes/genbank/bacteria/

3 http://202.120.12.135/TADB2/index.php

4 http://www.R-project.org/

5 http://www.ncbi.nlm.nih.gov/genbank/
